# Zebrafish Models to Study Ectopic Calcification and Calcium-Associated Pathologies

**DOI:** 10.3390/ijms24043366

**Published:** 2023-02-08

**Authors:** João M. A. Santos, Vincent Laizé, Paulo J. Gavaia, Natércia Conceição, M. Leonor Cancela

**Affiliations:** 1 Centre of Marine Sciences, University of Algarve, 8005-139 Faro, Portugal; 2 Faculty of Medicine and Biomedical Sciences, University of Algarve, 8005-139 Faro, Portugal; 3 Algarve Biomedical Center, University of Algarve, 8005-139 Faro, Portugal

**Keywords:** ectopic calcification, mineralization, zebrafish, calcium, disease modeling

## Abstract

Ectopic calcification refers to the pathological accumulation of calcium ions in soft tissues and is often the result of a dysregulated action or disrupted function of proteins involved in extracellular matrix mineralization. While the mouse has traditionally been the go-to model organism for the study of pathologies associated with abnormal calcium deposition, many mouse mutants often have exacerbated phenotypes and die prematurely, limiting the understanding of the disease and the development of effective therapies. Since the mechanisms underlying ectopic calcification share some analogy with those of bone formation, the zebrafish (*Danio rerio*)—a well-established model for studying osteogenesis and mineralogenesis—has recently gained momentum as a model to study ectopic calcification disorders. In this review, we outline the mechanisms of ectopic mineralization in zebrafish, provide insights into zebrafish mutants that share phenotypic similarities with human pathological mineralization disorders, list the compounds capable of rescuing mutant phenotypes, and describe current methods to induce and characterize ectopic calcification in zebrafish.

## 1. Introduction

Calcium is central to human physiology, being a major player in the homeostasis of mineralized tissues (e.g., bone and teeth), but also of soft tissues, where it is involved in blood clotting, muscle contraction, nerve function, and regulation of heart rhythm, among other processes [1,2]. Under pathological conditions, calcium may accumulate in soft tissues increasing their stiffness and affecting their function [3]. The pathological accumulation of calcium ions in soft tissues is defined as ectopic calcification and, while all soft tissues can be affected, vascular and cartilaginous tissues are among those most prone to calcium deposition leading to a pathological phenotype [4,5,6].

Ectopic calcification was long thought to be an aging-dependent disorder, but significant data has recently come forward indicating that it may also result from dysfunctional anti-mineralizing proteins [7,8]. In the latter case, circulating calcium in excess may precipitate, deposit, and accumulate in soft tissues. Deposits become progressively more crystalline and insoluble, hardening the tissue, and affecting its function [9]. Evidence of ectopic calcification at a young age is usually associated with a pathological condition, such as chronic kidney disease or autoimmune diseases [10,11].

Two distinct forms of ectopic calcifications have been described in humans. Metastatic calcification is characterized by an accumulation of calcium in soft tissues following an increase in serum levels, while dystrophic calcification results from an accumulation of calcium at pathologically altered sites [12]. Despite a better understanding of the pathologies behind disorders characterized by ectopic calcification, there is still a lack of efficient therapies capable of effectively preventing and treating ectopic mineral deposition [13].

Animal models of ectopic calcification are critical to better understand the disease pathophysiology and allow the screening of novel therapeutics [4]. While the mouse has often been the model of choice to investigate the mechanisms of ectopic calcification and has greatly contributed to a better understanding of calcium-associated pathologies [4,7], it has some issues that have prevented further advances in this field. When compared to many single-gene human diseases, loss-of-function mouse models often show exacerbated phenotypes and die prematurely, which limits our understanding of the pathological mechanisms [14,15]. For example, the matrix Gla protein (MGP) is a vitamin K-dependent protein present in the extracellular matrix and a well-documented inhibitor of ectopic mineralization, whose dysfunction is associated with the development of Keutel syndrome [16]. While only a few patients show vascular calcifications, the *Mgp* null mice die within the first two months of age due to extensive mineralization of arteries and subsequent rupture [17].

The zebrafish (*Danio rerio*) has emerged as a valuable model to gain insights into the mechanisms underlying the development of human diseases as it brings intrinsic advantages over mammalian models. Among those, it is worth mentioning that (i) a single breeding event can produce hundreds of embryos for testing, allowing a robust statistical analysis; (ii) embryos and larvae are optically clear and develop externally, allowing easy and direct visualization of the developmental process; (iii) amenability to genetic manipulation and availability of tools for genetic screening and editing; and (iv) a fast development throughout adulthood, allowing genetic experiments to be performed within a short period [18,19]. Zebrafish has gained momentum in the last decades for the study of bone disorders [20]—with tools and methodologies available to assess osteogenic and mineralogenic effects [21]—and, in the present review, we provide evidence that zebrafish is also a relevant model to study pathologies associated with tissue calcification. Based on the analysis of scientific articles referenced in the PubMed database “pubmed.ncbi.nlm.nih.gov (accessed on 7 December 2022)”, we also outline the current tools, techniques, and mutants available to gain insights into mechanisms of ectopic calcification and calcium-associated pathologies, and list compounds with the capacity to rescue mutant phenotypes and contribute to the next-generation therapeutics to prevent or treat ectopic calcification disorders.

## 2. Zebrafish In Vivo Models to Study Ectopic Mineralization Disorders

The successful modeling of ectopic mineralization disorders in zebrafish implies the conservation throughout vertebrate evolution of (i) the mechanisms underlying calcium metabolism, (ii) the sites of calcium-phosphate crystal (predominantly in the form of hydroxyapatite) deposition, and (iii) a phenotypic response that mimics human mineralization disorders.

Although calcium intake occurs in the kidney and intestine in mammals and the gills and yolk skin (during development) in fish, the major principles of calcium transport and hormonal control were found to be conserved from zebrafish to mammals [22,23]. For instance, a loss-of-function mutation in zebrafish Trpv6—a calcium channel expressed in the kidney and intestinal epithelia and central to calcium absorption in humans [24]—generated a 68% reduction in systemic calcium content and significantly inhibited calcium uptake in the yolk sac and gills in zebrafish embryos [25]. Similarly, adult zebrafish fed a diet rich in cholesterol develop lesions in the intimal layer region of the dorsal aorta and show macrophage infiltration mimicking the development of atherosclerosis and ectopic calcification observed in humans [12,13]. Similar pathological outcomes were observed in zebrafish mutants with impaired production of proteins important for lipid metabolism, such as the low-density lipoprotein (LDL) receptor [26], the lipoprotein lipase apolipoprotein C2 (APOC2) [27,28], or the cholesterol catabolism liver X receptor (LXR) [29].

Several zebrafish models of Mendelian genetic disorders share phenotypic similarities with the acquired forms of metastatic and dystrophic calcifications and may serve as genetically controlled systems to study human calcification disorders. Recent studies on small teleost fishes have allowed the identification of various genetic factors that contribute to ectopic calcification; they are further discussed below and summarized in Table 1.

### 2.1. Models of GACI and PXE 

Generalized arterial calcification of infancy (GACI)—caused by mutations in the gene *Ectonucleotide pyrophosphatase/phosphodiesterase 1* (*ENPP1*)—and pseudoxanthoma elasticum (PXE)—caused by mutations in the gene *ATP binding cassette subfamily C member 6* (*ABCC6*)—are rare autosomal recessive genetic disorders associated with arterial and cartilage calcification, and ectopic calcifications of elastic fibers, respectively [41,42]. Zebrafish *enpp1* mutants were shown to develop ectopic calcification of the skin, cartilage, heart, intracranial space, and notochord sheet, independently of osteoblast activity. They also displayed higher osteoclast activity at sites of ectopic calcification, and bisphosphonates could efficiently rescue associated phenotypes [30]. Zebrafish *abcc6a* mutants showed defects in vertebral calcification and displayed ectopic calcification in soft tissues [32]. Since Abcc6a is present in the eyes and heart of the zebrafish, extensive calcification of the ocular sclera and Bruch’s membrane and a fibrotic heart were also observed in the *abcc6a* mutants [33]. 

Vitamin K was proposed to be central to the pathophysiology of GACI and PXE as patients usually show a reduction in circulating vitamin K, which may be a countermeasure against pathological mineralization [43]. While the administration of vitamin K failed to rescue the ectopic calcification phenotype of *Abcc6* knockout mice [44], it restored mineralization levels and lowered the sites of ectopic mineralization in zebrafish *abcc6a* mutants [33,43]. Interestingly, treatment with warfarin, a vitamin K antagonist, exacerbated the ectopic mineralization phenotype in *abcc6a* zebrafish mutants [43]. Aberrant mineralization in PXE pathogenesis is partly due to excessive DNA damage. In this regard, zebrafish *abcc6a* mutants treated with minocycline, a DNA damage response inhibitor, had reduced occurrences of pathological mineralization [45].

### 2.2. Models of Chronic Kidney Disease 

Chronic kidney disease (CKD) patients are likely to suffer from ectopic calcifications due to a dysregulated mineral metabolism and pathological alterations in KLOTHO and Fibroblast Growth Factor (FGF) 23 [46]. Mice deficient for αKlotho or Fgf23 have age-related disorders, including abnormal mineral regulation and ectopic calcification [47,48]. As both mutants share similar phenotypes, a link between the two proteins was established, and αKLOTHO was found to function as a co-receptor of the FGF receptor and to be responsible for activating and controlling the production of FGF23 [49,50]. αKLOTHO and FGF23 are produced in the kidney and bone tissues, respectively, and both are central to mineral homeostasis. Changes in their expression are strongly associated with the development of chronic kidney disease (CKD) [51,52]. Brood stocks of *αKlotho* and *Fgf23* null mice are difficult to maintain as they die by three months of age; this represents a major bottleneck in the use of these murine models [47,48].

In zebrafish, *αklotho* expression is detected in the adult kidney and *fgf23* is continuously expressed in the corpuscles of Stannius, an endocrine gland close to the nephron that contributes to calcium homeostasis, where Fgf23 is responsible for adjusting and regulating calcium metabolism [53,54]. As for the mouse mutants, zebrafish mutants for *αklotho* and *fgf23* have a short lifespan [34]. However, the disease phenotype only occurs at approximately five months of age, later than in the mouse model (i.e., as soon as one month of age). Thus, zebrafish mutants can reach adulthood and reproduce, allowing the maintenance of a mutant brood stock, a major drawback of the mouse model. Both zebrafish mutants display ectopic calcification of the vessels throughout the body, especially in the outflow tract of the heart and the bulbus arteriosus, a pathological calcification likely associated with premature aging, ectopic activation of osteoclast differentiation, and age-associated vascular calcification [34]. Indeed, in vivo studies using *αklotho, fgf23,* and *ennp1* zebrafish mutants have consistently shown an increase in osteoclast activity around mineralized soft tissues, hinting at the existence of osteoclasts that develop as a response to ectopic calcifications [30,34,55,56]. 

### 2.3. Model of Primary Familial Brain Calcification

Primary familial brain calcification (PFBC) is a rare progressive neurodegenerative disorder characterized by bilateral brain calcifications and associated with symptoms of dementia, Parkinsonism, and dystonia [57,58]. In 2018, mutations in the gene *Myogenesis regulating glycosidase* (*MYORG)* have been linked to the development of autosomal recessive PFBC [59,60]. The central role of Myorg in the disease was confirmed by the observation of irregular bilateral brain calcifications in *Myorg* knockout mice at nine months of age [58]. Because the development of PFBC in the murine model requires a long period of time, alternative solutions were pursued, and zebrafish provided a timelier model. Zebrafish larvae where *myorg* expression was knocked-down using morpholinos (Table 1) exhibited multiple calcifications in the brain already at two days post-fertilization [60], demonstrating the suitability of zebrafish morphants over mouse mutants.

### 2.4. Other Models That Remain to Be Assessed

Mutations in the genes *golgin b1* (*golgb1*) and *activin A receptor, type 1 like* (*acvr1l*) have been associated with the development of hyperphosphatemic familial tumoral calcinosis (HFTC) [37] and fibrodysplasia ossificans progressiva (FOP) [39]—rare disorders associated with the development of ectopic calcification [61,62]. While there is no data showing that zebrafish mutants for *golgin b1* and *acvr1l* develop ectopic calcification, it is unknown whether these fish simply failed to do so, or it was not observed or reported as it was not the focus of these studies. So far, mutations in the zebrafish orthologs of many single-gene human diseases—associated with ectopic mineralization disorders—faithfully mimicked disease phenotype and show pathological calcium deposition. Therefore, it is likely that many zebrafish mutants for genes associated with calcium mineralization disorders—whose phenotype has yet to be published—may develop ectopic mineralization phenotypes. Zebrafish mutants that may develop ectopic calcifications, and are therefore promising targets for the development of new models, are summarized in Table 2.

## 3. Tools to Study Ectopic Calcification in Zebrafish

### 3.1. Genetic-Based Approaches to Develop Mutant Lines

Most zebrafish models initially used to study ectopic mineralization have been developed through forward genetic approaches. In a screening to identify novel regulators of osteogenesis and bone mineralization, a mutant named dragonfish (dgf) showed ectopic mineralization in the craniofacial and axial skeleton [63]. This mutant was later reported to have altered enpp1 expression and it remains today one of the most used and well-described models of ectopic calcification [30]. The Zebrafish Mutation Project, a large-scale N-ethyl-N-nitrosourea (ENU) mutagenesis project, has generated a mutant archive of over 40,000 alleles covering 60% of zebrafish protein-coding genes, with many mutants yet to be characterized [64]. In relation to this article, four zebrafish mutants for αklotho were identified and are available from zebrafish stock centers. Each mutant has an allele with one point mutant that induces a premature stop in four out of five exons. At this point, only αklotho^sa18644^ studies—targeting exon 3—have been reported and the phenotype closely matches the loss-of-αKlotho function previously reported in zebrafish [35].

Reverse genetics has also been successfully applied to zebrafish. Morpholino antisense oligomers (MOs) are the preferred method of gene knockdown in zebrafish [65,66] and the initial approach taken at generating ectopic calcification in zebrafish models. Injection of *abcc6a*-specific MOs in zebrafish eggs induced cardiac malformations and developmental defects in 8 dpf morphant larvae similar to those observed in GACI patients [67]. Similarly, myorg-specific MOs induced brain calcification in 2 dpf morphant larvae [40]. CRISPR/Cas9 genome-editing technology has also been successfully applied to zebrafish [68,69] making it possible to phenotype F0 generation zebrafish (founders), also known as crispants, within weeks [70]. As many zebrafish mutants are already available, CRISPR/Cas9 is a promising tool to accelerate the study of ectopic mineralization in situations in which (i) a mutant is not available; (ii) to investigate a gain of function; (iii) and to target important functional domains. 

### 3.2. Techniques for the Detection of Abnormal Calcium Deposition

The detection and quantification of calcium deposition in zebrafish can be achieved through a variety of techniques that are based on the optical detection of changes in luminescence, fluorescence, or absorbance of an organic indicator that specifically binds to calcium ions [71]. Several water-soluble dyes can be used to detect tissue calcification, calcein and alizarin red S—that emit green and red signals, respectively, when bound to calcium-based crystals—have been mostly used in zebrafish [72]. Both dyes can be used in vivo—e.g., live staining without the sacrifice of the animal—allowing the real-time detection of calcium deposition [73]. Repetitive alizarin red S staining performed at low concentrations can be applied to zebrafish without affecting bone growth and mineralization [74]. Furthermore, alizarin red S can be combined with alcian blue, a polyvalent basic dye that stains cartilage in blue, allowing the visualization of calcium deposition within cartilaginous tissues [74]. Calcein and alizarin red S have similar efficiency in staining calcium deposits, thus the choice of using either dye often depends on the usage of transgenic fish models to complement the fluorescent reporter protein. As most transgenic fish models use enhanced green fluorescent protein (eGFP) [75,76], alizarin red S staining is often preferred over calcein. 

Von Kossa’s staining is also a very popular method to detect the presence of abnormal deposits of calcium-phosphate crystals in the body. This histological method is based on the transformation of calcium phosphate salts into silver phosphate salts, which undergo photochemical degradation when illuminated with a UV light, leading to the formation of dark silver deposits [77]. While its first usage dates back to more than a century, von Kossa’s staining remains a widely used method to detect the presence of vascular calcification in human tissue samples [78,79].

### 3.3. Radiographic Methods

Histological techniques used to detect ectopic calcification have limitations associated with the physical sectioning of hard tissues that often result in tissue loss or incomplete tissue sectioning. In addition, visualization of calcium deposition in elongated structures (e.g., blood vessels) and in a single section is extremely difficult. Micro-computed tomography (micro-CT) is commonly used for the 3D imaging and analysis of skeletal structures and other calcified tissues [80,81]; it utilizes X-rays to create cross-sections of a physical object to render a 3D image. Traditional micro-CT scans have been a reliable method for visualization of calcified bone tissues in zebrafish but it has lacked the resolution and tissue contrast to detect ectopic calcification [82]. However, recent advances in micro-CT and high-resolution imaging have reached a point in which direct visualization of nanoscale structures and the detection of ectopic calcification sites are possible [83,84]. Similarly, Raman spectroscopy, which is used to distinguish changes in biomolecules (e.g., minerals) present in tissues [85], has already been applied to the diagnosis of human calcification disorders [86,87] and represents a promising tool for the detection of ectopic calcification in zebrafish embryos, as it allows both the visualization and profiling of calcium deposits across mineralizing and soft tissues [88,89].

### 3.4. Ectopic Calcification-Inducing Drugs

The identification of compounds that induce phenotypical changes similar to those observed in pathological conditions is of the utmost interest as these compounds allow quick, easy, and cost-effective access to in vitro or in vivo disease models. Although the precise mechanisms that lead to ectopic calcification remain to be better understood, molecules that disrupt the balance between pro- and anti-mineralizing pathways have often been associated with pathological mineralization [90,91].

**Figure 1:**
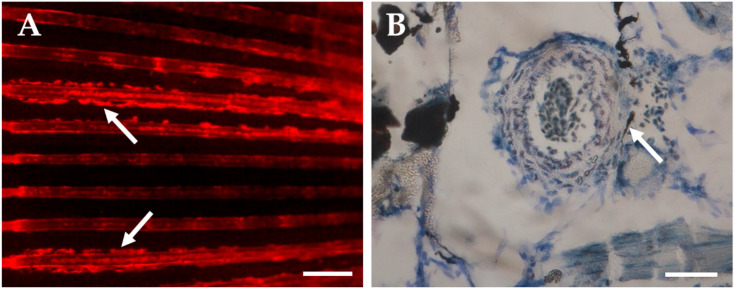
Vascular calcification in six-month-old zebrafish exposed for fifteen days to 25 mg/L of sodium warfarin. (**A**) Alizarin red S in vivo staining of calcified caudal fin blood vessels (white arrows) and (**B**) von Kossa’s staining of a calcified artery counterstained with toluidine blue (white arrow). Bar is 500 µm in (**A**) and is 50 µm in (**B**).

Vitamin K vitamers are central to bone mineralization through their role in the carboxylation of several vitamin K-dependent bone-related proteins, such as osteocalcin, matrix Gla protein, and Gla-rich protein [92,93,94]. Warfarin and other vitamin K antagonists inhibit the activity of vitamin K epoxide reductase and consenquently the recycling of vitamin K back to its active form. Sodium warfarin has been used in anticoagulation therapy in humans for seven decades [95,96], but long-term treatments have been associated with systemic arterial calcification [97,98], and warfarin is contraindicated during pregnancy as fetal exposure can lead to the development of warfarin embryopathy, a rare condition associated with abnormal bone and cartilage growth [99]. Mice treated with warfarin showed significant cardiovascular calcification that compromised cardiovascular function [100]. In zebrafish, embryonic exposure to warfarin has also been associated with ectopic calcification, and a decrease in γ-glutamyl carboxylase activity and embryonic lethality [101,102]. Long-term exposure of zebrafish to warfarin led to the development of warfarin embryopathy-associated phenotypes [101,103,104]. Similarly, adult zebrafish exposed to 25 mg/L of sodium warfarin for fifteen days developed vascular calcification, further supporting its suitability as an ectopic calcification-inducing drug (Figure 1).

### 3.5. Screening of Drugs to Rescue Pathological Calcification Disorders

Therapeutic strategies to efficiently counteract the accumulation of calcium ions in soft tissues responsible for ectopic mineralization disorders have yet to be developed. Antiresorptive drugs have been tried, but they were only able to delay and not prevent disease progression [105,106]. The inherent advantages and a similar response to anti-mineralizing compounds (Table 3)—when compared to mouse models—provide the proof-of-concept necessary to validate zebrafish as a fast high-throughput screening model for the identification of novel anti-mineralizing drugs.

## 4. Conclusions

**Figure 2:**
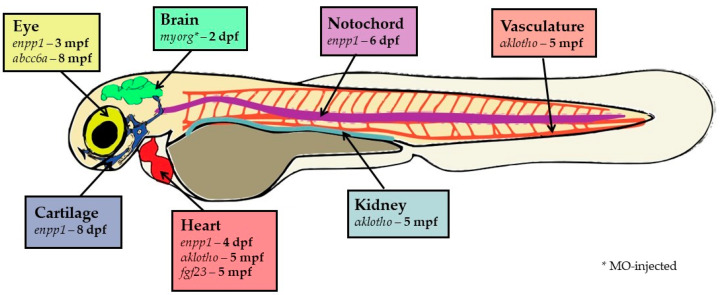
Current models that are available to study ectopic and soft tissue calcification in zebrafish. Information reported here includes target genes, target tissues, and the onset of ectopic calcification observed in zebrafish mutants. Representative image of a zebrafish larvae at 5 dpf. dpf, days post-fertilization; mpf, months post-fertilization; MO, morpholino oligomer. * Morpholino-injected.

Ectopic calcification disorders are medical conditions that affect the well-being and life expectancy of many people worldwide and for which therapeutics remain to be developed. To improve the current scenario, the complex mechanisms underlying these disorders must be better understood. Until recently, most of the information regarding pathological calcification was collected from human patients and mouse models, two biological systems with inherent limitations. Zebrafish models have recently emerged with the potential to provide new and fast insights into pathological mechanisms, as they can overcome some of these limitations (e.g., knockout mice being non-viable) and accelerate the collection of data with phenotypic alterations occurring at early stages of development. As for mammalian systems, zebrafish ectopic calcification models develop calcification in most soft tissues, and the detection of sites of ectopic calcification could be detected early during development (as soon as 2 dpf, the period at which zebrafish larvae hatch). Figure 2 summarizes the different zebrafish models available to study mechanisms of ectopic mineralization.

Thanks to the Zebrafish Mutation Project “zmp.buschlab.org (accessed on 7 December 2022)”, there are currently dozens of available mutants with genes associated with calcium mineralization disorders that remain to be analyzed and studied (Table 1) and eggs can be ordered from international repositories such as the Zebrafish International Resource Center (ZIRC), the European Zebrafish Resource Center (EZRC) or the China Zebrafish Resource Center (CZRC). Thanks to advances in gene-editing techniques, methods to induce and detect ectopic mineralization, and currently available mutant and transgenic lines, combined with some fish inherent advantages, the zebrafish has become the new go-to model organism to study the mechanisms of ectopic calcification and calcium-associated pathologies.

## Figures and Tables

**Table 1:** Zebrafish mutants to study ectopic mineralization disorders.

Name	Gene *	Description	ZFIN ID	Human Disease	Reference
enpp1^hu4581/hu4581^ (*dragonfly, dgf*)	*enpp1*	Pyrophosphatase, role in the regulation of bone mineralization	ZDB-GENE-040724-172	Generalized arterial calcification of infancy; Pseudoxanthoma elasticum; Autosomal recessive hypophosphatemic rickets	[30]
abcc6a^hu4958/hu4958^ (*gräte, grt*)	*abcc6a*	Transmembrane transporter, role in the regulation of bone mineralization	ZDB-GENE-050517-18	Generalized arterial calcification of infancy; Pseudoxanthoma elasticum	[31,32,33]
kl^zf3212/zf3212^ sa18644	*kl*	Anti-aging hormone, role in the regulation of mineral homeostasis	ZDB-GENE-110221-1	Hyperphosphatemic familial tumoral calcinosis-3	[34,35]
fgf23^zf3214/zf3214^	*fgf23*	Growth factor, role in calcium ion homeostasis	ZDB-GENE-050201-4	Autosomal recessive hypophosphatemic rickets; Hyperphosphatemic familial tumoral calcinosis-2	[34]
golgb1^bsl077/bsl077^	*golgb1*	Membrane trafficking in protein’s secretory pathway	ZDB-GENE-030429-9	Hyperphosphatemic familial tumoral calcinosis	[36,37]
MO2-acvr1l MO4-acvr1l zf1073Tg	*acvr1l*	Activin A receptor, type 1	ZDB-GENE-990415-9	Fibrodysplasia ossificans progressiva	[38,39]
myorg-E2I2-MO myorg-ATG-MO	*myorg*	Putative glycosidase	ZDB-GENE-091113-62	Primary familial brain calcification	[40]

* Gene name and acronym: *enpp1*, *ectonucleotide pyrophosphatase/phosphodiesterase 1*; *abcc6a*, *ATP-binding cassette*, *sub-family C (CFTR/MRP)*, *member 6a*; *kl*, *klotho*; *fgf23*, *fibroblast growth factor 23*; *golgb1*, *golgin B1*; *acvr1l*, *activin A receptor*, *type 1 like*; *myorg*, *myogenesis regulating glycosidase (putative)*.

**Table 2:** Zebrafish mutants yet to be investigated.

Name	Gene *	Description	ZFIN ID	Human Disease
la028295Tg sa8734	*nt5e*	5′-nucleotidase, role in hereditary arterial/articular calcification syndrome	ZDB-GENE-040426-1261	Calcification of joints and arteries
sa37832	*ankha*	Inorganic pyrophosphate transport regulator	ZDB-GENE-050913-33	Chondrocalcinosis 2
sa12038 sa32626	*slc34a2a*	High-affinity inorganic phosphate:sodium symporter	ZDB-GENE-000524-1	Pulmonary alveolar microlithiasis
la022442Tg sa37585	*slc34a2b*	High-affinity inorganic phosphate:sodium symporter	ZDB-GENE-030709-1	Pulmonary alveolar microlithiasis
sa9319	*gnas*	GTPase	ZDB-GENE-090417-2	Osseous heteroplasia, progressive
sa39971	*mgp*	Inhibitor of vascular mineralization	ZDB-GENE-060928	Keutel syndrome
sa12462	*samd9l*	Inflammatory response and the control of extra-osseous calcification	ZDB-GENE-130530-738	Normophosphatemic familial tumoral calcinosis
sa41932	*fam20a*	Golgi-associated secretory pathway pseudokinase	ZDB-GENE-081022-117	Enamel renal gingival syndrome
sa20589	*casr*	Parathyroid hormone secretion and renal tubular calcium re-absorption regulator	ZDB-GENE-050119-8	Familial hypocalciuric hypercalcemia syndrome

* Gene name and acronym: *nt5e*, *5′-nucleotidase, ecto (CD73)*; *ankha*, *ANKH inorganic pyrophosphate transport regulator a*; *slc34a2a/b*, *solute carrier family 34 member 2a/b*; *gnas*, *GNAS complex locus*; *mgp*, *matrix gla protein*; *samd9l, sterile α motif domain containing 9 like*; *fam20a*, *FAM20A golgi associated secretory pathway pseudokinase*; *casr, calcium-sensing receptor*.

**Table 3:** Compounds to rescue ectopic mineralization in zebrafish.

Compound	Mutant	Rescue Effect	Reference
Etidronate (100 µM)	*enpp1^−/−^*	Rescues aspects of the *dgf* phenotype	[30]
	*abcc6a^−/−^*	Reduced spinal mineralization	[107]
Vitamin K1 (80 µM)	*enpp1^−/−^*	Reduces hypermineralization	[43]
	*abcc6a^−/−^*		[43,107]
Sodium thiosulfate (20 µM)	*abcc6a^−/−^*	Reduced spinal mineralization	[107]
Magnesium citrate (10 mM)	*abcc6a^−/−^*	Reduced spinal mineralization	[107]
Minocycline (3 µM)	*abcc6a^−/−^*	Reduced aberrant mineralization	[45]

## Data Availability

Not applicable.
